# A novel oscillometric method for blood pressure measurement with reduced cuff pressure and measurement time

**DOI:** 10.1038/s41440-026-02676-8

**Published:** 2026-05-13

**Authors:** Yuki Ota, Mitsuo Kuwabara, Kazuomi Kario

**Affiliations:** 1 Division of Cardiovascular Medicine, Department of Medicine, Jichi Medical University School of Medicine, Tochigi, Japan; 2 Omron Healthcare Co. Ltd., Kyoto, Japan

**Keywords:** Blood pressure monitoring, Oscillometric, Intra-cuff pressure, Measurement time, Digital hypertension

## Abstract

The oscillometric method is the predominant technique for non-invasive blood pressure (BP) measurement worldwide, and has enabled widespread adoption of home BP monitoring, which is crucial for hypertension management. However, limitations in the usability of BP monitors, and particularly the pain and discomfort caused by excessive cuff inflation, can discourage consistent daily use of home BP monitors. To address this, we developed a novel oscillometric-based technology that reduces maximum cuff pressure (MaxCP) and measurement time while maintaining accuracy. We validated the performance of the novel method using a pre-existing dataset of oscillometric waveforms by comparing the estimated BP values with reference values obtained using the conventional method. The calculated BP values demonstrated high agreement with the conventional method, with intraclass correlation coefficients of 0.98 (systolic BP) and 0.94 (diastolic BP). Furthermore, the novel method reduced average MaxCP by 38.1 mmHg and measurement time by 6.3 s. These findings suggest that our approach can significantly enhance the user experience in BP monitoring without compromising measurement accuracy, potentially improving adherence to daily monitoring routines.

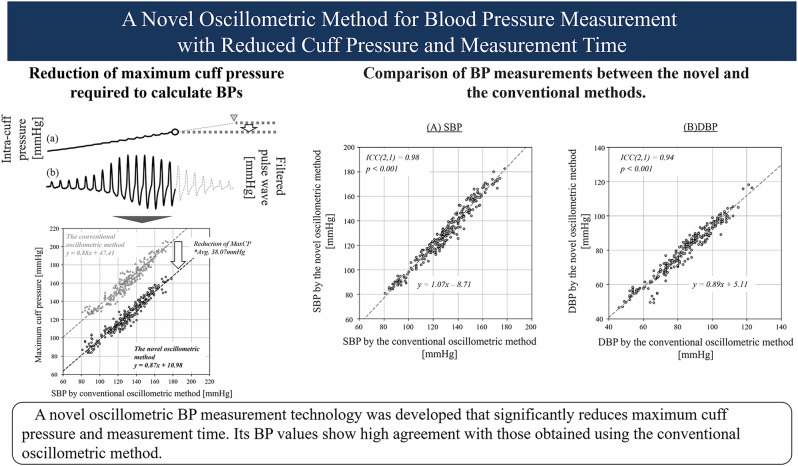

## Introduction

Automated blood pressure (BP) measurement based on the oscillometric method is utilized worldwide as a non-invasive BP measurement method [1, 2]. A BP monitor (BPM) using the oscillometric method can automatically calculate systolic BP (SBP) and diastolic BP (DBP) for each subject, based on characteristics of the pulse-wave signal extracted from cuff pressure during measurement [3]. If the BPM is validated according to appropriate clinical validation protocols, users can obtain BP readings as accurate as those by the gold standard auscultation method, without any special skills or knowledge. This technological innovation has enabled hypertensive patients to measure their out-of-office BPs without professional supervision and has contributed to the establishment of new hypertension management indicators, such as home BP and ambulatory BP.

Among these out-of-office BPs, a large amount of evidence has been accumulated regarding the clinical significance of home BP [4, 5]. Therefore, the home BP-guided approach has become increasingly important in hypertension management and is recommended in clinical practice. A key clinical advantage of home BP is its strong ability to predict future cardiovascular events. In addition to capturing BP values with minimal white-coat effect, home BP measurement can evaluate the average value of multiple daily readings, which contributes to its high prognostic value [6]. Furthermore, daily BP measurement in hypertensive patients has been reported to increase awareness of their health status, which may in turn improve medication adherence and ultimately BP control [7].

With the aim of promoting the use of home BP monitoring-based hypertension management in clinical practice, we have long been engaged in development of this measurement modality, with a focus on enhancing accuracy and usability [8]. As part of this effort, we developed a function that estimates the user’s SBP during BP measurement and automatically controls the optimal maximum cuff pressure (MaxCP), thereby preventing unnecessary cuff inflation. Although this function can reduce the burden of BP measurement, further improvement is needed because MaxCP can still exceed 200 mmHg in subjects with very high BP. Such excessive cuff pressure may cause discomfort and pain, which could discourage patients from measuring their BP as often as recommended in current guidelines. This undermines the advantage of home BP monitoring—namely, the ability to obtain daily BP information. One of the technologies with potential for solving this issue is cuffless BP estimation technology, which circumvents the requirement of cuff inflation [9, 10]. This technology estimates BP values from physical quantities other than pressure information, such as pulse transit time (PTT) and the shape of the pulse wave obtained by photoplethysmography (PPG). Since it does not require cuff compression, it is expected to realize non-invasive monitoring of continuous BP values. However, its accuracy in tracking BP changes due to medication or during sleep has not been adequately demonstrated, and therefore it is not currently recommended for use in clinical hypertension practice [1, 2]. Taking the above into consideration, we developed a novel technology based on the cuff-oscillometric method, which has been clinically validated and is recommended in hypertension guidelines, that dramatically reduces the burden associated with cuff inflation. We expect that this technology will reduce the pain and discomfort caused by cuff pressure during BP measurement, and thereby encourage hypertensive patients to continue measuring their BP daily.

In this paper, we describe the technical methodology underlying our novel oscillometric-based technology and present the results of a comparative validation study of BP values obtained with the new method and with the conventional oscillometric method (conventional method). Furthermore, we evaluate how much this technology reduces BP measurement time and maximum cuff pressure compared with the conventional method.

## Materials and methods

### Study design

This study compares the SBP, DBP, MaxCP, and measurement time between our novel oscillometric method (novel method) and the conventional method. To compare the two methods, we used a dataset acquired with an existing BPM (HEM-7600T; Omron Healthcare Co., Ltd.) for validation study [11]. The original study that collected this dataset was approved by the institutional review boards of the Biwako Central Hospital (BCH-144) and Omron Healthcare Co., Ltd. (IRB-1552). The present secondary analysis was approved by the HURECS.org ethics review board (E2026-04-001).

The BP based on the conventional method was the value displayed by the existing BPM, and MaxCP and measurement time were the values recorded by the device at each measurement. The measurement time was defined as the period from the first pulse wave detection by the existing BPM to the end of each measurement process. The novel method used the intra-cuff pressure waveform stored in the dataset to simulate BP values, MaxCP, and measurement time, with the conventional-method BP values masked. In addition, MaxCP and measurement time were evaluated after classifying subjects into two groups based on SBP: high blood pressure (SBP ≥ 135 mmHg) and normal blood pressure (SBP < 135 mmHg) groups.

### Data source and participants

We used a dataset acquired in a previous validation study with an existing BPM [11]. In that study, 101 participants were screened; 16 were excluded based on prespecified eligibility criteria, leaving 85 participants (38 men [45%] and 47 women [55%]). The mean age was 50.4 ± 11.7 years (range, 25–80 years), and the mean arm circumference was 31.2 ± 5.5 cm (range, 22.1–41.4 cm). The arm-circumference categories were as follows: 22.0–32.0 cm, 50.0%; 32.1–42.0 cm, 48.8%; 22.0–27.0 cm, 32.6%; and 37.1–42.0 cm, 20.9%. The dataset includes raw intra-cuff pressure waveforms and the conventional-method outputs—i.e., BP values, maximum cuff pressure (MaxCP), and measurement time—recorded by the existing BPM.

### Conventional oscillometric method

BP values based on the conventional method obtained by existing BPM using the cuff-oscillometric method are calculated from the pulse-wave signal extracted from the cuff pressure during measurement. Two approaches are used to calculate BP: one estimates BP during cuff inflation [12], and the other during cuff deflation after inflation [3]. Because our novel technology is based on BP estimation during cuff inflation, we describe here the cuff-oscillometric method that measures BP during cuff inflation.

**Fig. 1:**
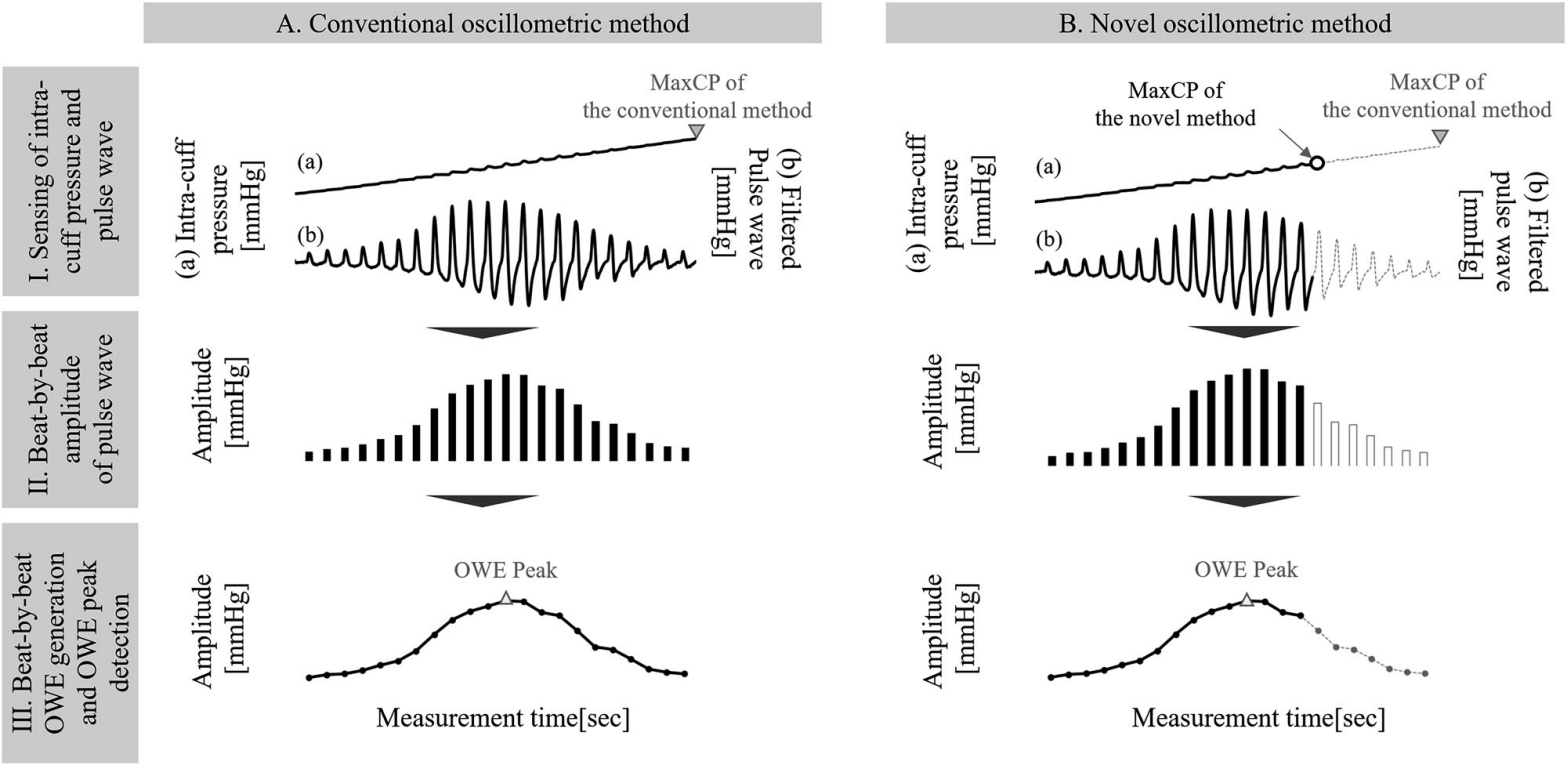
Schematic comparison of the conventional and novel oscillometric methods during cuff inflation. **A** The conventional method inflates the cuff to a maximum cuff pressure (MaxCP) set well above the peak of the oscillometric waveform envelope (OWE). **B** In contrast, the novel method terminates inflation immediately after detecting the OWE peak, resulting in a significantly lower MaxCP

Figure. 1A shows the processes of extracting the pulse-wave signal and calculating the BPs using the conventional method. Once the BPM starts BP measurement, it automatically inflates the cuff while continuously measuring the intra-cuff pressure (Fig. 1A, Ia). The intra-cuff pressure is then digitally filtered to generate a filtered pulse wave (Fig. 1A, Ib). The BPM will automatically terminate inflation when the cuff pressure reaches the MaxCP. In the next phase, an amplitude is extracted from this filtered pulse wave for each beat (Fig. 1A, II). The amplitude of each pulse wave is determined by calculating the difference between the maximum and minimum values of the filtered pulse wave. By combining the amplitudes obtained by each beat, a bell-shaped oscillometric waveform envelope (OWE) is constructed (Fig. 1A, III). The peak of this OWE represents the time point when the pulse amplitude caused by oscillation in cuff pressure reaches its maximum. Finally, once the prerequisites for calculating BP are met, the SBP and DBP are calculated based on the generated OWE shape.

### Novel oscillometric method

Figure. 1B depicts the BP calculation process using our novel method; the key difference from the conventional method is the criterion for terminating cuff inflation. In the novel method, cuff inflation is terminated in real time once the OWE amplitude is judged to have passed its peak. This is achieved by an algorithm that monitors the continuous change in pulse-wave amplitudes and identifies the point at which the amplitude stops increasing and begins to decrease. The process of extracting the pulse-wave signal during inflation is the same as for the conventional method. To facilitate comparison of the two methods, Fig. 1B indicates results for the conventional method as gray dotted lines and those for the novel method as black solid lines. The significant reduction in MaxCP achieved by the novel method, compared to the conventional method, can be clearly seen by comparing these lines.

**Fig. 2:**
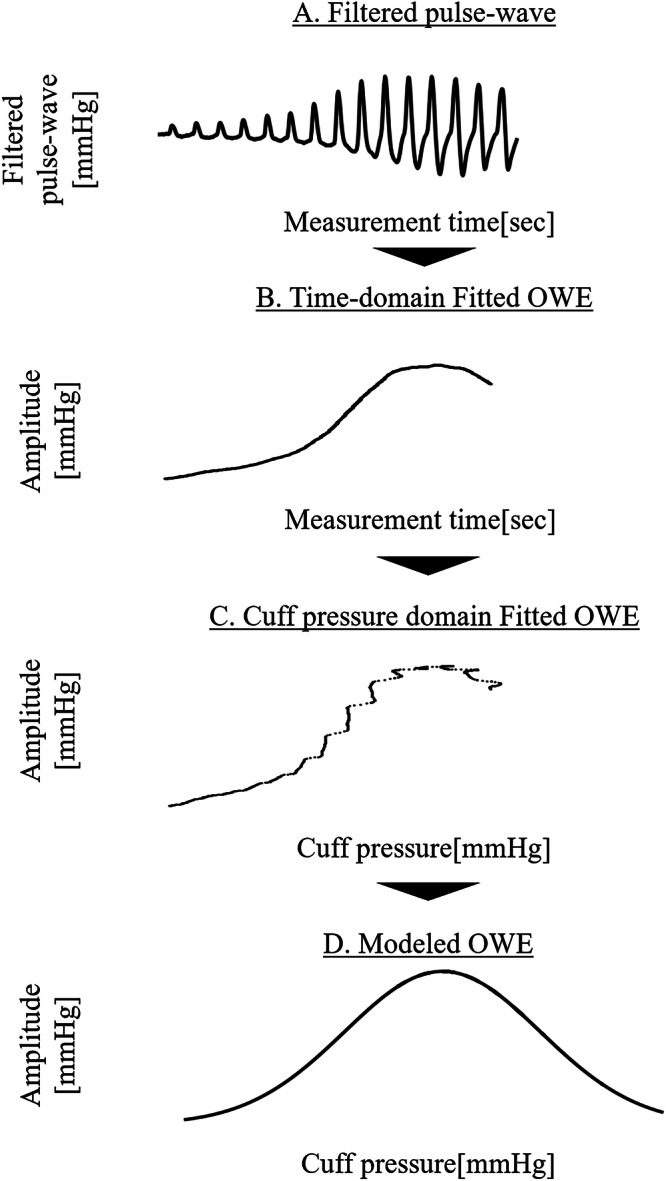
Reconstruction of the oscillometric waveform envelope (OWE) for the novel method. The figure shows the four-step process used to reconstruct a complete OWE from the limited dataset acquired by the novel method. **A** The filtered pulse wave is obtained during continuous cuff inflation. **B** The time-domain fitted OWE is obtained by fitting the OWE model to the measured amplitudes of the filtered pulse wave. **C** The fitted OWE is replotted in the cuff-pressure domain (cuff pressure, rather than time, on the horizontal axis). **D** The resulting model is used to reconstruct a complete, bell-shaped OWE (black line) for BP calculation. BP blood pressure, OWE oscillometric waveform envelope

In the novel method, the reduction in MaxCP results in an incomplete construction of the OWE by terminating intra-cuff pressure earlier than with the conventional method (Fig. 1B, III). Therefore, BP cannot be calculated using the conventional method. To overcome this challenge, the novel method utilizes the pulse-wave signal acquired during a limited inflation phase to estimate the OWE. Constructing a complete OWE from a limited OWE requires three steps. First, the filtered pulse wave obtained during cuff inflation (Fig. 2A) is processed by convolution to generate the Time-domain fitted OWE (Fig. 2B). Second, the OWE is re-plotted against cuff pressure-domain (Fig. 2C). Third, the developed model equation is applied to this cuff pressure-domain fitted OWE. This equation is based on a function designed to represent the typical bell shape of the OWE. Using a limited set of measured OWE datapoints, we estimate the function parameters, such as the peak position, height, and width, thereby generating a modeled OWE that closely matches the measured OWE (Fig. 2D).

Although it is possible to calculate BP using the smoothed modeled OWE, an important challenge remains. A single modeled OWE cannot fully represent the diversity of OWEs across individuals. For example, the OWE in a subject with high arterial stiffness differs from that in a subject with normal arterial stiffness, so a generic mathematical model may not fully reproduce the individual’s actual physiological state. As a result, the calculated BP may be inaccurate.

To address this challenge, the novel method enables accurate calculation of BPs by adding a post-processing step that adjusts the modeled OWE to reflect characteristics of the subject’s waveform. The process extracts multiple features from the pulse wave and OWE, such as the degree of discrepancy between the fitted and modeled OWE, and the shape of the pulse wave. By quantifying these features and complementing the smoothed modeled OWE with a modeled OWE, the novel method corrects the calculated BP. Therefore, even if the amount of information is reduced by significantly reducing MaxCP, an accurate BP can be calculated.

### Statistical analysis

Agreement between the BP values from the novel and conventional methods was assessed using scatterplots and intraclass correlation coefficients (ICCs). MaxCP and measurement time were compared and evaluated using Welch’s T-test.

## Results

### High agreement between BP values of the novel and conventional methods

**Fig. 3:**
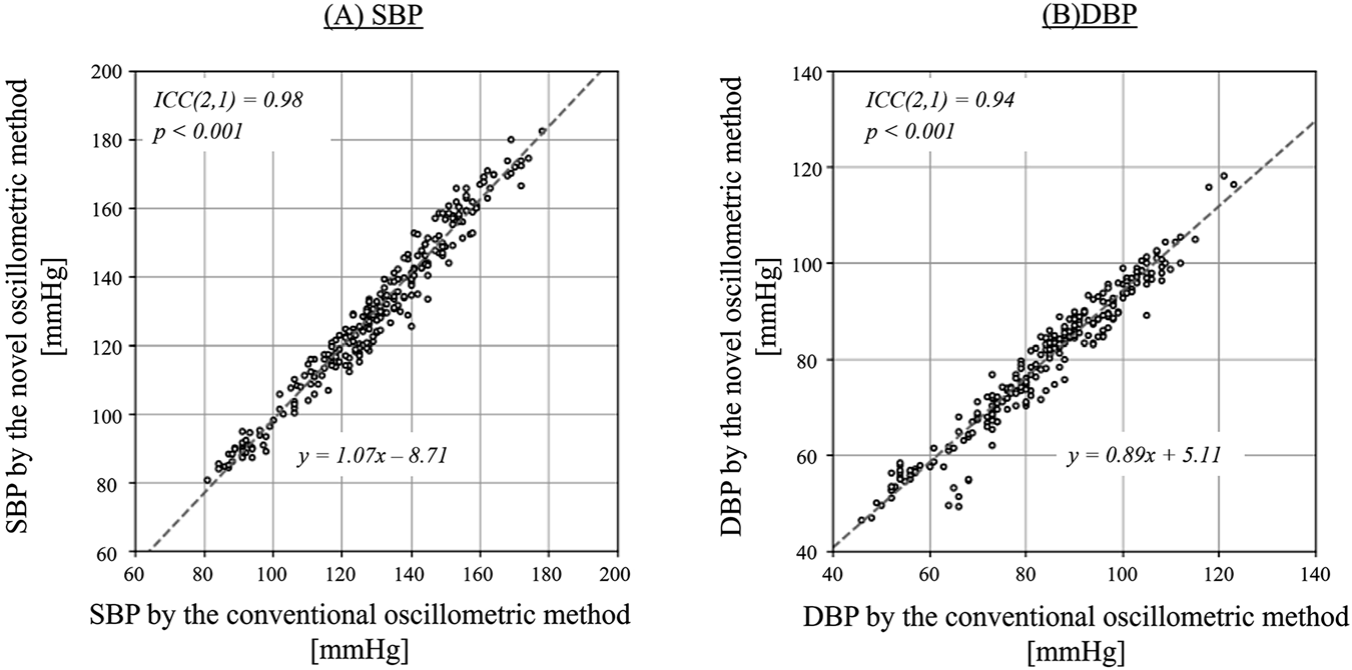
Comparison of BP measurements between the novel and conventional methods. Scatterplots compare the BP values obtained with the novel method versus the conventional method for all 255 measurements. **A** Comparison of SBP between the two methods. **B** Comparison of DBP between the two methods. The solid line in each plot represents the regression line. BP blood pressure, SBP systolic BP, DBP diastolic BP, ICC intraclass correlation coefficient

Figure 3A, B shows the scatterplots of SBP and DBP obtained by the novel and conventional methods, respectively. The ICCs between the two methods were 0.98 for SBP and 0.94 for DBP (both *p* < 0.001). A significantly high degree of agreement was observed between the methods, demonstrating that the novel method, despite using less cuff pressure pulse wave information to derive SBP and DBP, can calculate BP values with accuracy comparable to that of the conventional method.

### Reduction in MaxCP by the novel method

**Fig. 4:**
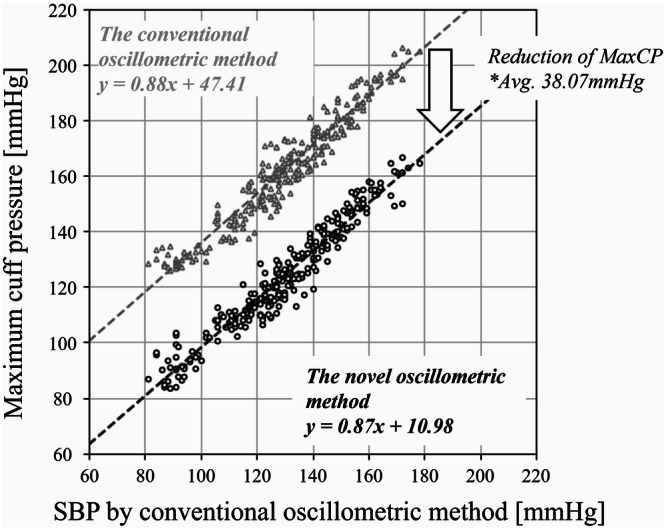
Relationship between SBP and MaxCP for the novel and conventional methods. This scatterplot shows the MaxCP required by the conventional method (gray triangles) and the novel method (black circles) as a function of the reference SBP. The solid lines represent the linear regression for each dataset. The plot visually demonstrates that the novel method required a substantially lower MaxCP across the entire range of SBP. BP blood pressure, SBP systolic BP, MaxCP maximum value of cuff pressure

Figure 4 shows the scatterplot of SBP obtained by the conventional method and the MaxCP for each subject obtained using the novel (black circles) and conventional (gray triangles) methods. In the conventional method, the regression equation is y = 0.88 x + 47.41, and MaxCP is on average 32.2 mmHg higher than SBP for each subject. On the other hand, in the novel method, the regression equation is y = 0.87 x + 10.98, and MaxCP is on average 5.9 mmHg lower than SBP for each subject. In this verification study, 89.0% of subjects (*n* = 255) had a MaxCP lower than their SBP. The mean ± standard deviation (SD) for MaxCP was 161.6 ± 19.6 mmHg with the conventional method and 123.5 ± 19.3 mmHg with the novel method, corresponding to an average reduction of 31.3% (38.1 mmHg) in MaxCP with the novel method compared with the conventional method.

In the subgroup analysis, the mean MaxCP reduction ( ± SD) was 37.8 ± 6.6 mmHg in the hypertensive group (*n* = 104) and 38.3 ± 7.3 mmHg in the non-hypertensive group (*n* = 151). A Welch’s t-test showed no significant difference in the mean reduction between the two groups (t(234) = -0.55, *p* = 0.59). These results demonstrate that the reduction in MaxCP achieved by the novel method is not dependent on the BP level.

### Reduction in measurement time by the novel method

**Fig. 5:**
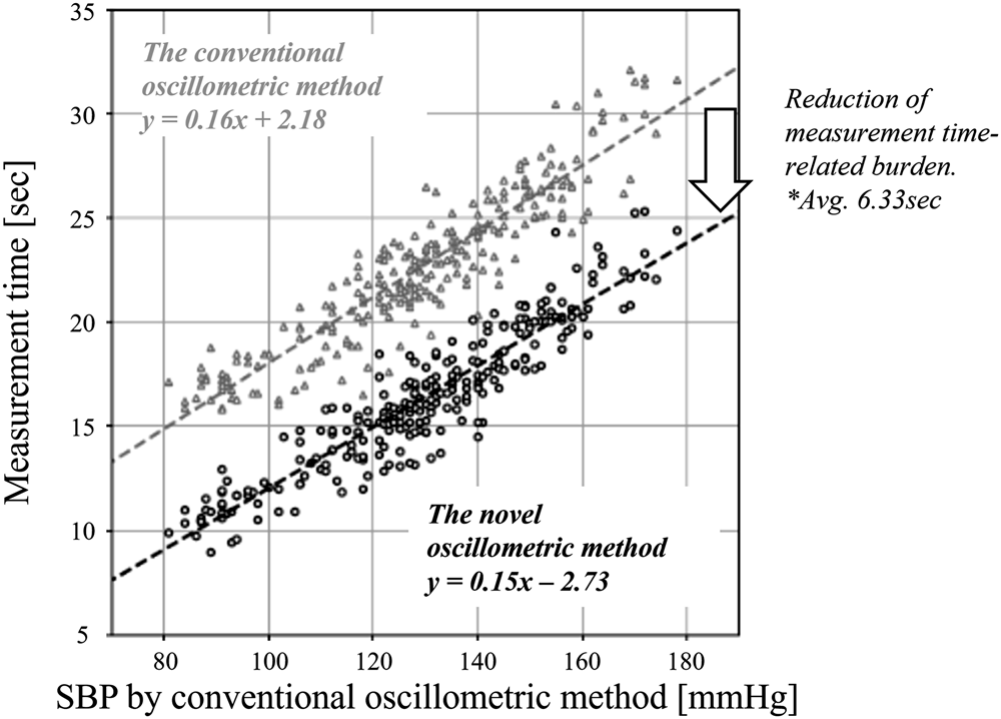
Relationship between SBP and measurement time for the novel and conventional methods. The measurement time is defined as the time from when the first cuff pressure pulse wave is observed to when cuff inflation ends. This scatterplot shows the measurement time required by the conventional method (gray triangles) and the novel method (black circles) as a function of the reference SBP. The solid lines represent the linear regression for each dataset. The plot visually demonstrates that the novel method was consistently faster across the entire range of SBP. SBP systolic blood pressure

Figure 5 shows the scatterplot of SBP obtained by the conventional method and the measurement time for each subject using the novel method (black circles) and the conventional method (gray triangles). Here, the measurement time is defined as the time from when the first cuff pressure pulse wave is observed to when cuff inflation ends. The regression equations were y = 0.16x + 2.18 and y = 0.15x – 2.73 for the conventional and novel methods, respectively. The mean measurement time ( ± SD) was 22.6 ± 3.7 s for the conventional method and 16.3 ± 3.4 s for the novel method. This corresponds to an average reduction of 6.3 s with the novel method compared with the conventional method.

In the subgroup analysis, the mean reduction ( ± SD) in measurement time was 6.5 ± 1.1 s in the hypertensive group (*n* = 104) and 6.2 ± 1.2 s in the non-hypertensive group (*n* = 151). The difference was statistically significant by Welch’s t-test (t(235) = 2.46, *p* < 0.05). This result indicates that the novel method is more effective in reducing BP measurement time in subjects with high BP levels.

## Discussion

In this study, we developed a novel BP measurement technology based on the oscillometric method that can significantly reduce the maximum cuff pressure and measurement time. BP values estimated by the novel method via simulation using cuff-pressure waveforms acquired with an existing BPM closely agreed with the BP values reported by the BPM using the traditional oscillometric method. This technology adopts the general concept of the oscillometric method, which calculates BP values from the characteristics of arterial pressure pulse wave information during cuff inflation, but uses a groundbreaking technology to predict the entire envelope using less cuff pressure pulse-wave information than required by the conventional method.

### Technical advances of the novel method

The main advantage of the novel method is that it can terminate cuff inflation earlier than the conventional method because it estimates the full OWE from a limited portion of the cuff-pressure pulse-wave data, rather than requiring oscillation data collected across the entire pressure range, as in the conventional method. In addition to the modeled OWE, the novel method extracts features such as the shape of the pulse wave during inflation and uses them as correction factors. By analyzing waveform features associated with BP and selecting those that most improved estimation accuracy, we achieved performance comparable to the conventional method even with limited signal information. Two technological advancements are expected to be important for future implementation in BPMs and broader clinical adoption.

The first technological advance involves the pumps required for cuff inflation, and the microcontroller unit (MCU). The innovation in pump performance has improved the signal-to-noise ratio of the obtained pulse-wave signal, thereby enabling the utilization of more detailed signal features than previously possible. While it was challenging to ensure sufficient accuracy with BP measurements when estimating BP from an OWE derived from limited information, our new approach achieved accuracy comparable to that of the conventional method. This was accomplished by inputting features extracted from the high-quality pulse-wave signal, such as its shape, its amplitude, and their rate of change during inflation.

Supporting the improved pump performance, an advance in MCU performance made it possible to process various signal features in parallel during cuff inflation. This capability is crucial, as the novel method requires real-time calculation of multiple features in addition to cuff control and OWE estimation. Without parallel processing, these intensive calculations would significantly prolong the measurement time. Therefore, the enhanced MCU performance is critical for implementing the novel method while maintaining user comfort. It is worth noting that these performance improvements were achieved without increasing the size of the components, which may eventually allow the novel method to be implemented with a basic BPM.

The second technological advance is our unique analytical approach. In the novel method, various pulse-wave features are used as correction factors when calculating BP, so selecting effective features for this purpose was essential. We made several hypotheses about pulse-wave features that are thought to be related to BP values, and thoroughly analyzed which of these features would contribute significantly to improving the accuracy of BP measurement. In the future, by further reducing the number of functions to the minimum required to maximize performance, it may be possible to reduce the software to a size that can be implemented in existing BPMs.

### Expected clinical benefits of the novel method

This novel method is expected to have several advantages in clinical practice. First, it may reduce the impact of stress-induced fluctuations in BP by alleviating the pain or discomfort caused by cuff inflation during BP measurement, which can increase BP through sympathetic nervous system activation [13]. This would be especially advantageous for nighttime BP measurement during sleep. It is well known that nighttime BP is one of the most important predictors of cardiovascular disease, and the measurement and management of nighttime BP are important [14, 15]. However, it has been reported that measurements using the conventional oscillometric method, which completely occludes the blood vessel, may cause sleep disturbances due to cuff inflation, and such sleep disturbances would in turn impact the BP values being recorded. By reducing sleep disturbances, the novel method may minimize their impact on nighttime BP measurement.

Another expected benefit is the reduction of physiologically induced BP fluctuations that occur when BP is measured repeatedly over short time intervals. After the blood vessels are fully occluded by cuff inflation, creating a transient ischemic state, vasodilatory substances are released when the cuff is deflated, resulting in a vasodilation response [16]. This phenomenon is known as flow-mediated dilation (FMD). To minimize this effect, a time interval of 1–2 min between measurements is recommended, but this is not always strictly followed in clinical practice. By avoiding complete occlusion of blood vessels, the novel method may attenuate the vasodilation response even when the recommended measurement interval is not observed, yielding BP measurements with greater prognostic value.

### Future prospects

For this novel method to be adopted in clinical practice, its measurement accuracy must be evaluated against established validation standards, such as ISO81060-2:2018. In addition, the extent to which the cuff-inflation burden (e.g., maximum cuff inflation and measurement time) can be reduced relative to the conventional oscillometric method should be quantified. Finally, further studies will be needed to confirm the anticipated clinical benefits described above.

## Conclusion

We have developed a novel BP measurement technology based on the oscillometric method that can significantly reduce the maximum cuff pressure and measurement time while maintaining BP measurement accuracy equivalent to that of the conventional method. This new technology has the potential to dramatically reduce the burden on subjects caused by BP measurements and contribute to improving compliance with daily BP monitoring.
